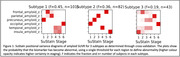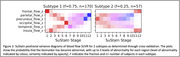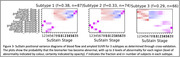# Improved Disease Subtyping and Staging using Dual Biomarker Amyloid PET

**DOI:** 10.1002/alz.088749

**Published:** 2025-01-09

**Authors:** Catherine J Scott, William Coath, John C Dickson, Sarah J McQuaid, David M Cash, Jonathan M Schott, the Insight 46 team

**Affiliations:** ^1^ Dementia Research Centre, UCL Queen Square Institute of Neurology, London UK; ^2^ Institute of Nuclear Medicine, University College London Hospitals, London UK; ^3^ Dementia Research Centre, UCL Queen Square Institute of Neurology, University College London, London UK; ^4^ Barts Health NHS Trust, London, London UK

## Abstract

**Background:**

Data‐driven disease progression models of Alzheimer’s disease (AD) have identified subtypes in regional patterns of Aβ deposition using amyloid PET. In addition to Aβ accumulation, early frame measures of tracer delivery from amyloid PET are strongly correlated with blood flow. This work explores whether combining tracer delivery with amyloid binding measures can improve the subtype and stage characterisation over amyloid binding alone.

**Methods:**

Data was included from participants in three studies: Insight46, a neuroimaging sub‐study of the Medical Research Council National Survey of Health and Development (n=405), AVID2, a study of patients with a differential diagnosis of Alzheimer’s Disease (n=15), and YOAD, a study of patients with clinically diagnosed young onset Alzheimer’s disease (n=15). All participants underwent a full dynamic 18F‐Florbetapir PET/MR scan. PET images were reconstructed from 0‐2 and 40‐50 minutes post injection to generate the tracer delivery and amyloid binding images, respectively. T1 parcellations were used to calculate regional standardised uptake value ratios (SUVRs) using a cerebellar grey matter reference region. Tracer delivery and amyloid binding SUVRs for 6 cortical regions were converted into z‐scores, assuming unimodal and bimodal distributions respectively, and modelled using subtype and stage inference (SuStaIn). Participants fitted as stage zero were excluded.

**Results:**

Figure 1 shows the three subtypes found using regional amyloid binding SUVR. Subtype 2 accumulates amyloid first in the occipital lobe, while for subtypes 1 and 3, which accumulate Aβ first in the precuneus and insula respectively, this is the last area to show Aβ deposition. Two distinct subtypes were found with blood flow SUVR; frontal or precuneus first blood flow reduction, Figure 2. When both measures are combined, Figure 3, the data show that subtypes 1 and 2 exhibit amyloid accumulation before blood flow changes, whereas subtype 3 shows blood flow changes in all regions first. The two blood flow subtypes are spread across all three combined subtypes, suggesting that each amyloid subtype may have several blood flow subtypes.

**Conclusions:**

Combining blood flow and amyloid SUVR within SuStaIn reveals new patterns of disease progression compared to amyloid alone. This may help us better understand heterogeneity in aging and disease.